# IL-11 promotes the treatment efficacy of hematopoietic stem cell transplant therapy in aplastic anemia model mice through a NF-κB/microRNA-204/thrombopoietin regulatory axis

**DOI:** 10.1038/emm.2017.217

**Published:** 2017-12-08

**Authors:** Yan Wang, Zhi-yun Niu, Yu-jie Guo, Li-hua Wang, Feng-ru Lin, Jing-yu Zhang

**Affiliations:** 1 Department of Hematology, The Second Hospital of Hebei Medical University, Hebei Key Laboratory of Hematology, Shijiazhuang, China

## Abstract

Hematopoietic stem cell (HSC) transplantation could be of therapeutic value for aplastic anemia (AA) patients, and immunosuppressants may facilitate the efficiency of the procedure. As anti-inflammatory cytokine interleukin-11 (IL-11) has a thrombopoietic effect, its use in cases of chronic bone marrow failure, such as AA, has been proposed to induce HSC function. However, the putative mechanisms that may support this process remain poorly defined. We found that decreased miR-204-5p levels were coincident with increased proliferation in mouse HSCs following exposure to IL-11 *in vitro*. Through inhibiting NF-кB activity, miR-204-5p repression was demonstrated to be a downstream effect of IL-11 signaling. miR-204-5p was shown to directly target thrombopoietin (TPO) via sequence-dependent 3′-UTR repression, indicating that this microRNA-dependent pathway could serve an essential role in supporting IL-11 functions in HSCs. Increased TPO expression in HSCs following IL-11 exposure could be mimicked or blocked by inhibiting or overexpressing miR-204-5p, respectively. Consistent with these *in vitro* findings, IL-11 promoted HSC engraftment in a mouse model of AA, an effect that was attenuated in cells overexpressing miR-204-5p. The reduction in miR-204-5p levels is an integral component of IL-11 signaling that may play an essential role in treating AA.

## Introduction

Aplastic anemia (AA) is a severe disease characterized by bone marrow failure and is associated with defective hematopoietic stem cell (HSC) functions.^1^ Normally, the limited lifespan of circulating blood cells necessitates a constant hematopoiesis that relies on self-renewing multipotent HSCs. These cells are maintained by a specialized microenvironment in the bone marrow, and, if needed, can give rise to a hierarchy of lineage-committed progenitor cells for massive expansion and differentiation into mature blood cells.^2^ Following the destruction of HSCs, inadequately replenished hematopoietic cells, including red blood cells, white blood cells and platelets, render patients with AA at a high risk of bleeding and infection. While the causes of the disease are incompletely understood, AA covers a wide spectrum of heterogeneous disorders that can be inherited or acquired^3^ and are characterized by multiple, potentially overlapping, symptom dimensions. Generally speaking, the hematopoietic stem cell compartment is challenged and subsequently destroyed by an aberrant immune response, which is primarily mediated by an expansion of cytotoxic T-cells (CTLs).^4, 5^ As such, patients with AA are often treated through immunosuppressive therapy to eradicate pathogenic T-cell clones, and this treatment is performed in combination with bone marrow transplantation.^6^ Although HSC transplantation can effectively improve the bone marrow function of patients with AA,^1, 7, 8^ their response rate and long-term survival still depend on the correction of immune irregularities. It is clear that rescuing the immunologically stressed and depleted stem cell compartment, such as by inhibiting the CTL reaction, should serve an important role in preventing the relapse or ultimate curing the disease.

Under the inflammatory conditions in AA, HSCs are directly or indirectly attacked by a plethora of dysregulated immune responses. The clonal expansion of CTLs is coupled to profound changes in the immune system, an effect that is mediated by abnormal T helper cells, T regulatory cells and Th17 cells.^1, 6^ For example, it has been described that the numbers of CD4^+^CD25^+^ FOXP3^+^ T cells are decreased in AA, and these cells have been implicated in defective immune homeostasis in the bone marrow.^9^ De Latour^10^ reported that the increased frequency and total number of CD3^+^CD4^+^ IL-17-producing T cells are partly responsible for marrow failure in patients with AA. In particular, Th1 lymphocytes, especially interferon (IFN)-γ-producing CD4^+^ T cells, play an important role in the pathogenesis of AA. Increased Th1 cell numbers were observed in patients with acquired AA,^11^ an effect that resulted in a shift towards a type-1 response. In turn, this response likely induces the death of HSCs and blocks their proliferation potential.^12^ Overall, studies have characterized the clinical relevance of type 1 immune reactions in patients with acquired AA, and the corresponding therapeutic strategy has begun to take advantage of the down-regulation in Th1 cells to reverse the loss of HSCs.^13, 14^

Interleukin-11 (IL-11) is known for its anti-inflammatory functions. For example, recombinant IL-11 can ameliorate inflammatory diseases, such as psoriasis,^15^ heart disease,^16^ periodontal disease^17^ and Crohn’s disease.^18^ Moreover, IL-11 can modulate cytokine production from activated Th1 cells and diminish the polarization of the type 1 response.^19^ These results suggest that IL-11 likely has a general therapeutic potential in diseases where the Th1 responses are essential. Indeed, low-dose IL-11 administration in pilot studies was shown to increase platelet counts without notable toxicity in thrombocytopenic patients with bone marrow failure.^20, 21^ Intriguingly, the unique IL-6 family cytokine also has well-defined actions in stimulating various stages and lineages of hematopoiesis.^22^ This protein can work synergistically with other cytokines to promote the proliferation, differentiation and commitment of multi-lineage progenitor cells in hematopoietic compartments. In the current study, we hypothesized that IL-11 may act directly on hematopoietic stem cells, a characteristic that could be exploited to treat patients with AA. Mechanistically, we focused on the microRNA (miRNA, miR) pathway downstream of IL-11 signaling, which may link the efficacy of IL-11 treatment with HSC transplantation, in a mouse model of AA.

## Materials and methods

### Isolation and culturing of mouse hematopoietic stem cells

Eight-week-old male C57/BL6 mice were used in this study, which followed the protocol approved by the Committee on the Ethics of Animal Experiments of The Second Hospital of Hebei Medical University. Mouse hematopoietic stem cells (HSC) were isolated and cultured as previously described.^23, 24^ Briefly, whole bone marrow was isolated from femurs and tibias of mice and suspended at a concentration of 1 × 10^8^ cells per ml. The cells were then incubated on ice for 20 min with a cocktail of APC-conjugated antibodies specific to mature lineages of hematopoietic cells (including CD5/Ly-1, CD45R/B220, CD11b/Mac-1, Ly-6G/Gr-1, Ter-119/Ly-76, CD8a/Ly-2, and CD4/Ly-4, eBioscience, San Diego, CA, USA), FITC-conjugated anti-mouse CD34, PE-conjugated anti-mouse c-Kit, and PE-conjugated Cy™7 anti-mouse Sca-1 (BD Pharmingen, San Diego, CA, USA). The CD34^−^LSK population (Lin^−^ Sca-1^+^ c-kit^+^) was then sorted with a MoFlo cell sorter (Dako, Santa Clara, CA, USA) and subsequently cultured with Iscove’s modified DMEM medium supplemented with 10% fetal calf serum,, 1% bovine serum albumin, 20 μM 2-β-mercaptoethanol, 2 mM glutamine and 1% streptomycin/penicillin at 37 °C in 5% CO_2_. IL-11 was obtained from R&D Systems and was used to treat the BM-HSC cells at 10 ng ml^−1^ for 24 h in the *in vitro* experiments, according to a previous report.^25^ The cultured cells were also characterized for HSC surface markers with PeCy5-conjugated anti-mouse CD48, APC-conjugated anti-mouse CD244 (eBioscience), and PE PE-conjugated anti-mouse antibodies CD150 (BioLegend, San Diego, CA, USA). The identity of the HSC population was confirmed as CD150^+^CD244^−^CD48^−^.

### BrdU incorporation assay

The BrdU assay was performed using a BrdU Cell Proliferation Assay Kit (Roche, Penzberg, Upper Bavaria, Germany), according to the manufacturer’s instructions. Briefly, the cells were incubated with BrdU-labeling medium for 1 h. After a PBS rinse, the cells were fixed by ethanol for 20 min at room temperature. An anti-BrdU working solution was then added, and the cells were incubated for 30 min at 37 °C. The cells were then washed before adding substrate solution, and the signal was measured in a microplate reader at an absorbance 450 nm.

### Quantitative real-time PCR

Total RNA was isolated with TriFast (PeqLab). The following primers were used: *TPO,* forward, 5′-ACC AAC TCC AGT GTC TCA G-3′ and reverse, 5′-TCC TTG TGT CCC GTT CAG-3′ *GAPDH,* forward,5′-AAG GTC ATC CCA GAG CTG AA-3′ and reverse, 5′-CTG CTT CAC CAC CTT CTT GA-3′. Triplicate PCR reactions were performed using a SYBR green-based system (Applied Biosystems, Waltham, MA, USA), and the expression levels were normalized to those of GAPDH.

### Western blot

Total cellular protein was prepared using RIPA lysis buffer and quantified using a Bio-Rad protein assay. The proteins were then separated by SDS-PAGE gel and transferred onto nitrocellulose membranes. The blots were incubated with anti-TPO, anti-p65 or anti-GAPDH antibodies purchased from Cell Signaling Technology at 4 °C overnight. The bands were visualized with Pierce ECL Substrate (Thermo Fisher Scientific, Waltham, MA, USA).

### Luciferase reporter assay

The wild type or mutated promoter region of miR-204-5p was cloned into the upstream promoter region of a pGL3-enhancer plasmid; the wild type or mutated 3′-UTR of mouse TPO mRNA was cloned into the open reading frame. The cell transfection was performed using the Neon Transfection system (Thermo Fisher Scientific). After transfection, the cells were allowed to grow for another 24 h. The luciferase activities were determined using a dual-luciferase reporter assay kit (Promega, Madison, WI, USA).

### Chromatin immunoprecipitation assay

HSCs were cross-linked with 1% formaldehyde and immunoprecipitated with an anti-p65 antibody (Cell Signaling Technology, Danvers, MA, USA) or IgG as a negative control. Eluted DNA from the immunoprecipitation was analyzed by quantitative PCR using PCR primers that were designed to amplify the promoter regions of miR-204. The binding efficiency was calculated as a ratio of amplification efficiency of the p65-IP sample to that of the IgG sample.

### MicroRNA profiling

To identify the putative regulatory microRNAs in HSCs following IL-11 treatment, we first screened miRNAs based on the 3′-UTR of mouse TPO gene using the miRanda algorithm.^26^ The resultant miRNAs were selected for a miRNA microarray with a customized probe set containing oligos (IDT) that were designed based on the miRNA registry database. Total RNA, including miRNA, was isolated from cultured cells at the indicated conditions using TRIzol (Invitrogen, Carlsbad, CA, USA) and was labeled as first-strand biotin-cDNA. The hybridization steps were performed on a hybridization station (Tecan) followed by an indirect detection of streptavidin-Alexa647 conjugate (Invitrogen). The microarray image analysis was carried out using GenePix Pro software (Molecular Devices, Sunnyvale, CA, USA).

### MicroRNA measurement, mimic and inhibitor assays

The expression levels of miR-204-5p were measured using TaqMan Advanced miRNA Assay kit (478491_mir; Thermo Fisher Scientific). The MISSION Lenti-microRNA Inhibitor kit against mouse miR-204-5p (MLTUD0124) and the negative control (HLTUD001C) were purchased from Sigma-Aldrich (St Louis, MO, USA). The shMIMIC mouse lentiviral miR-204-5p mimic (VSM6213-246434487) was purchased from GE Life Sciences. All of the lentiviral vectors were packaged and transduced into hematopoietic stem cells according to the manufacturer’s instructions.

### Mouse model of AA and HSC transplantation

All of the mice were injected subcutaneously in the dorsal region with 1500 mg kg^−1^ benzene in corn oil daily for 3 weeks. The mice were then randomly separated into various conditions (20 mice per group), followed by sham or HSC transplantation with different treatments (cells that received no treatment, or cells that stably expressed either miR-NC or miR-204-5p). For the HSC transplantation, the BM-HSCs were harvested, resuspended in sterile PBS and injected into the right femur, as previously described.^27^ Briefly, the mouse knee joint area was exposed by opening the skin above the patella. A 25G needle was used to make an intra-femoral tunnel at the end of femur, followed by injection of 5 μl of cell suspension (5 × 10^5^) into the bone marrow cavity through a Hamilton micro-syringe with a needle tip. For the IL-11 administration, the mice were injected subcutaneously with recombinant IL-11 (25 μg kg^−1^), according to previous reports,^28, 29, 30^ or control vehicle. The injections were performed twice daily, from 1 to 7 days after the transplantation. At 15, 30 and 45 days after the transplantation, whole blood was collected and diluted in Turk’s solution (1:20) for leukocyte (WBC) quantification with a hemocytometer, which was performed under a light microscope. Platelet quantification was performed in diluted blood with 1% ammonium oxalate (1:200). The hemoglobin concentrations were determined using the cyanmethemoglobin method, and the death of mice during the procedures was documented.

### Statistical analysis

All of the data in graphs are expressed as the means±s.d. as indicated. Two-tailed Student's *t*-tests, one-way analysis of variance (ANOVA) or two-way ANOVA were performed to compare the differences between cell conditions, as appropriate. Two-way ANOVA were used to evaluate the data for statistical comparison for the animal studies. *P*<0.05 indicates significance.

## Results

### IL-11 is a positive regulator of HSC self-renewal

**Figure 1:**
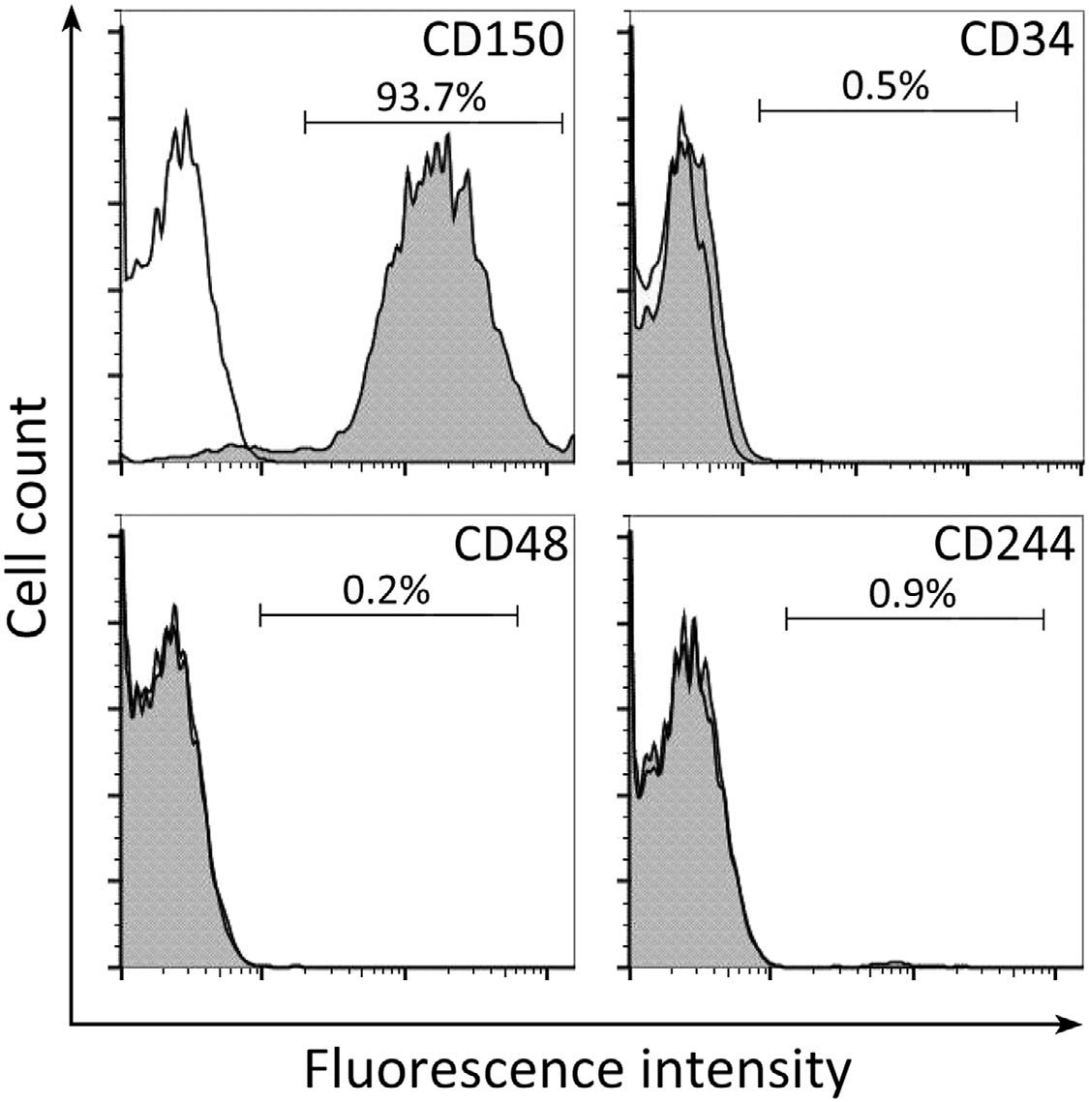
Isolated hematopoetic stem cells (HSCs) were positive for CD150 and negative for CD34, CD48 and CD244. HSCs were analyzed by flow cytometry for the cell surface markers CD150, CD34, CD48 and CD244 (gray), with positivity being defined relative to the signal from respective isotype controls (white).

**Figure 2:**
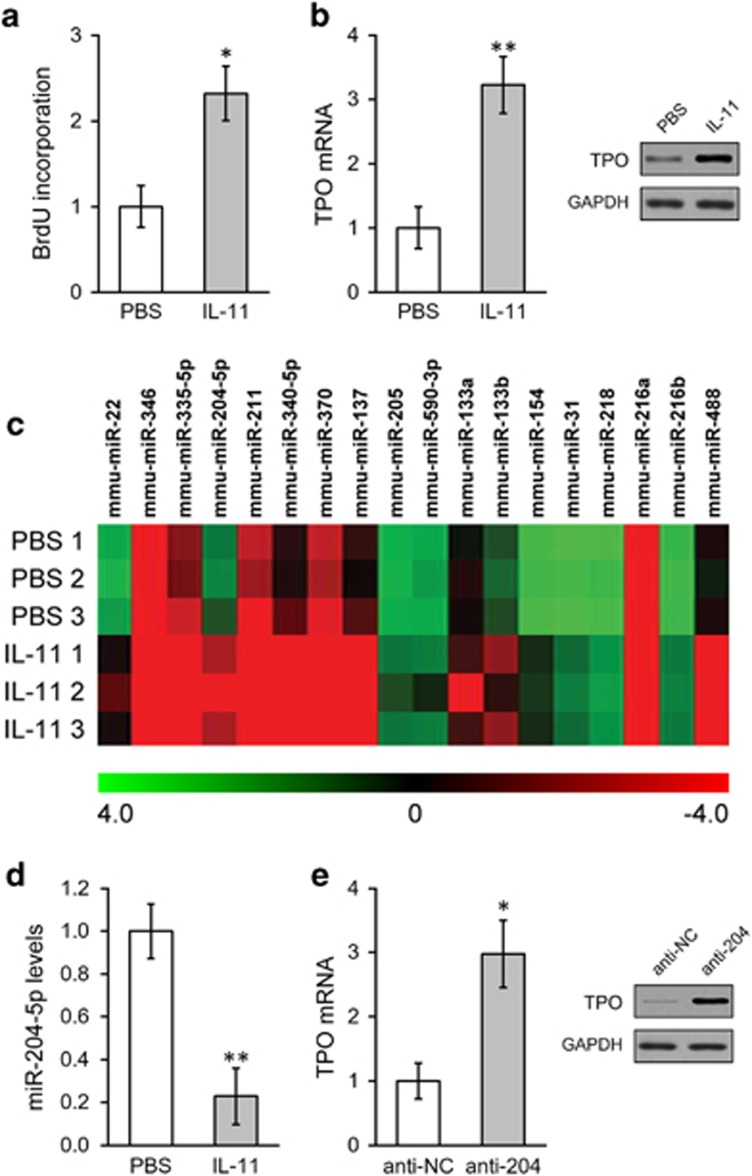
IL-11 up-regulates TPO expression by down-regulating miR-204-5p in hematopoetic stem cells (HSCs). (**a**) The proliferation of HSCs treated with either phosphate-buffered saline (PBS) or IL-11 was analyzed using a BrdU incorporation assay. (**b**) The mrna and protein levels of TPO in HSCs treated with either PBS or IL-11. (**c**) Eighteen miRNAs predicted to target TPO were analyzed by microarray following IL-11 treatment in HSCs. (**d**) The expression levels of miR-204-5p were analyzed using a TaqMan Advanced miRNA Assay in HSCs treated with either PBS or IL-11. (**e**) The mrna and protein levels of TPO in HSCs expressing either a negative control inhibitor (anti-NC) or a miR-204-5p inhibitor (anti-204). The values are shown as the mean±s.d. from three independent experiments. **P<0.01, *P<0.05 versus PBS or and anti-NC control.

To investigate the effects of IL-11 on HSCs, we first isolated the HSC population (CD34^−^LSK cells as Lin^−^ Sca-1^+^ c-kit^+^) from mouse bone marrow using a conventional method.^23^ The identity of cultured bone marrow derived (BM)-HSC cells was also confirmed by surface markers, including CD150 positivity and CD34, CD48 and CD244 negativity (Figure 1). Following the treatment of the BM-HSC cells with IL-11 at 10 ng ml^−1^ for 24 h, the *in vitro* proliferation of the HSCs was significantly increased, as determined using a BrdU incorporation assay (Figure 2a). In addition, the expression of thrombopoietin (TPO) in HSCs was greatly enhanced by IL-11 treatment, as shown by mRNA analysis, which was further confirmed at the protein level (Figure 2b).

### MiR-204-5p suppression mediates the TPO induction by IL-11 in BM-HSCs

We then tested the hypothesis that miRNAs might be involved in TPO stimulation by IL-11 in BM-HSCs. Using the miRanda algorithm,^26^ 18 miRNAs were predicted to be capable of recognizing sequences in the 3′-UTR of mouse TPO mRNA. The expression levels of several of these miRNAs were altered by IL-11 treatment in HSCs compared to the PBS treatment controls, as shown by a miRNA array assay (Figure 2c). Among these miRNAs, we confirmed using a miRNA assay that miR-204-5p was significantly downregulated by IL-11 treatment in HSCs (Figure 2d). Additionally, as shown in Figure 2e, upon a blockade of miR-204-5p function using a specific miR-204-5p inhibitor (anti-204), we found that TPO expression in HSC was significantly increased both at the mRNA and protein levels.

**Figure 3:**
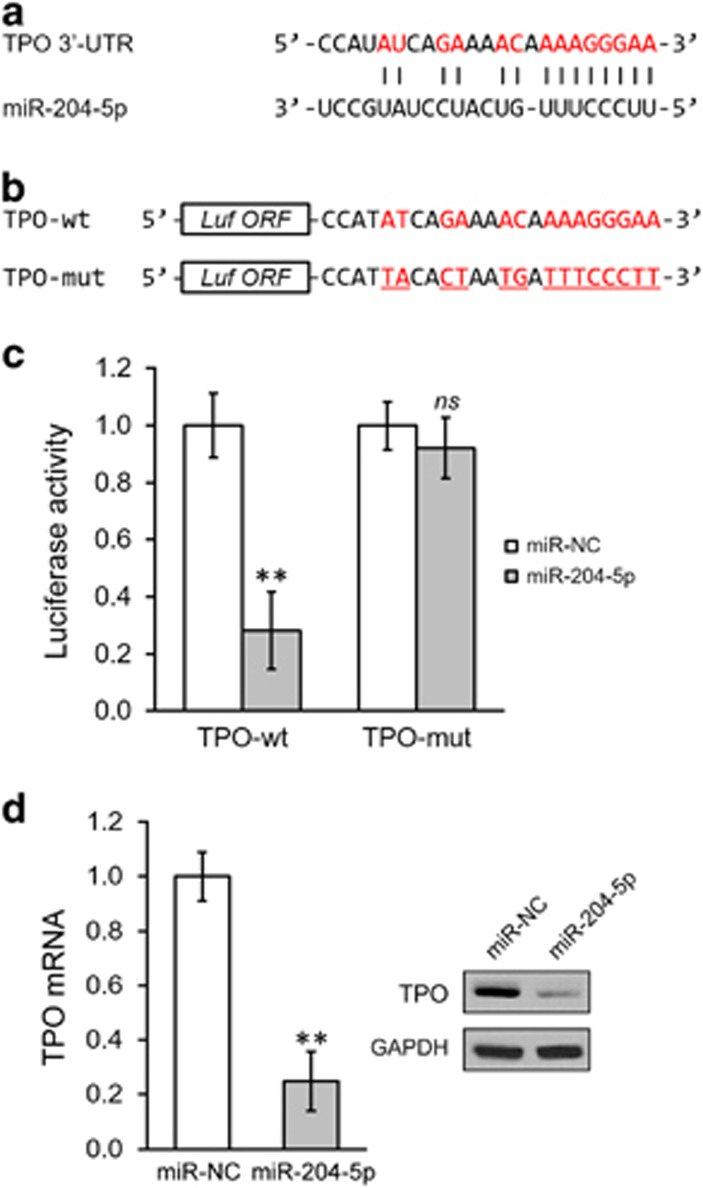
MiR-204-5p directly targets the 3′-UTR of TPO mRNA. (**a**) The sequences of the predicted miR-204-5p targeting sites on the 3′-UTR of TPO mRNA. (**b**) Wild type (TPO-wt) or mutated (TPO-mut, red underlined) sequences from the TPO mRNA 3′-UTR were cloned downstream of luciferase reporter gene (Luf ORF), as illustrated. (**c**) The luciferase activities of TPO-wt and TPO-mut constructs were determined in hematopoetic stem cells (HSCs) expressing miR-NC or miR-204-5p. (**d**) The mrna and protein levels of TPO in HSCs stably expressing either a negative control miR (miR-NC) or miR-204-5p. The values are shown as the mean±s.d. from three independent experiments. **P<0.01, NS, not significant, versus PBS or miR-NC control.

As the 3′-UTR of TPO mRNA contains a sequence that could potentially hybridize with miR-204-5p (Figure 3a), we tested the effect of miR-204-5p on TPO expression using a 3′-UTR luciferase reporter assay. We generated luciferase reporter constructs containing either the a wild type (TPO-wt) or mutated (TPO-mut) 3′-UTR of TPO mRNA (Figure 3b). To determine whether the activities of these reporters were regulated by miR-204-5p, we co-transfected these constructs into HSCs together with either a miR negative control (miR-NC) or miR-204-5p. As shown in Figure 3c, we observed a remarkable reduction of luciferase activity by miR-204-5p only in the reporter with the TPO-wt sequence, whereas this effect was not observed for the mutated TPO sequences (TPO-mut). These data suggested a sequence-dependent TPO repression by miR-204-5p via an interaction in the 3′-UTR. Consistent with this finding, the overexpression of miR-204-5p in HSCs resulted in a significant reduction of TPO both in mRNA and protein levels (Figure 3d). Together, our data suggest IL-11 could promote TPO expression in HSCs, and the IL-11-induced decrease in miR-204-5p levels might be specifically responsible for this process.

### NF-кB repression is required for miR-204-5p-dependent IL-11-mediated induction of TPO induction in HSCs

**Figure 4:**
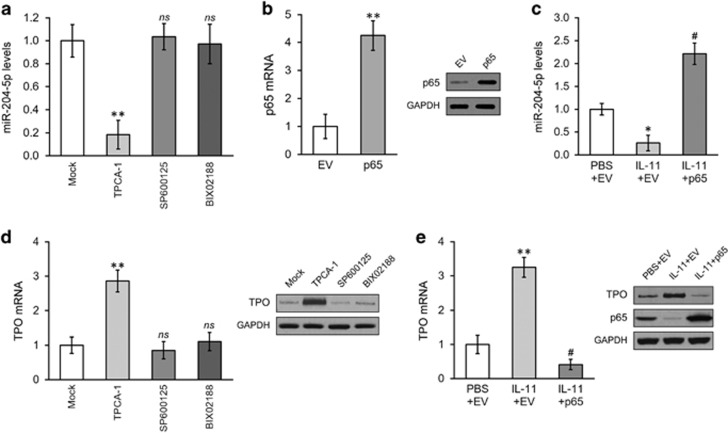
IL-11 inhibits NF-кB activation to downregulate miR-204-5p in hematopoetic stem cells (HSCs). (**a**) The expression levels of miR-204-5p in HSCs were analyzed following mock treatment or treatment with the IKK inhibitor TPCA-1, the JNK inhibitor SP600125 or the p38 MAPK inhibitor BIX02188. (**b**) The mRNA and protein levels of NF-кB in HSCs expressing either empty vector control (EV) or a plasmid overexpressing the NF-кB p65 subunit (p65). (**c**) The expression levels of miR-204-5p in HSCs following treatments with PBS+EV, IL-11+EV or IL-11+p65. (**d**) The mRNA and protein levels of TPO were examined in HSCs following a mock treatment or treatment with the IKK inhibitor TPCA-1, the JNK inhibitor SP600125 or the p38 MAPK inhibitor BIX02188. (**e**) mRNA and protein levels of TPO, as well as p65 protein levels, in HSCs following treatments with PBS+EV, IL-11+EV or IL-11+p65. The values are shown as the mean±s.d. from three independent experiments. **P<0.01, *P<0.05, NS, not significant versus mock, EV or PBS+EV. #*P*<0.05 versus PBS+EV and IL-11+EV.

We next investigated which IL-11 regulated process is responsible for the inhibition of miR-204-5p. We first examined the miR-204-5p levels of HSCs in the presence of TPCA-1 (IKK inhibitor), SP600125 (JNK inhibitor) or BIX02188 (MAPK inhibitor). As shown in Figure 4a, miR-204-5p expression was reduced only by TPCA-1, suggesting the involvement of the NF-кB pathway. We then overexpressed the NF-кB subunit p65 in HSCs (Figure 4b) and assessed whether the NF-кB pathway was indeed involved in IL-11-induced miR-204-5p suppression. As expected, p65 overexpression efficiently rescued the miR-204-5p inhibition after IL-11 treatment (Figure 4c). On the other hand, TPCA-1 treatment directly increased TPO expression both at the mRNA and protein levels (Figure 4d), mimicking the effect of IL-11. In addition, IL-11 treatment directly down-regulated p65 expression, whereas overexpressing p65 attenuated IL-11-mediated TPO induction in HSCs (Figure 4e).

**Figure 5:**
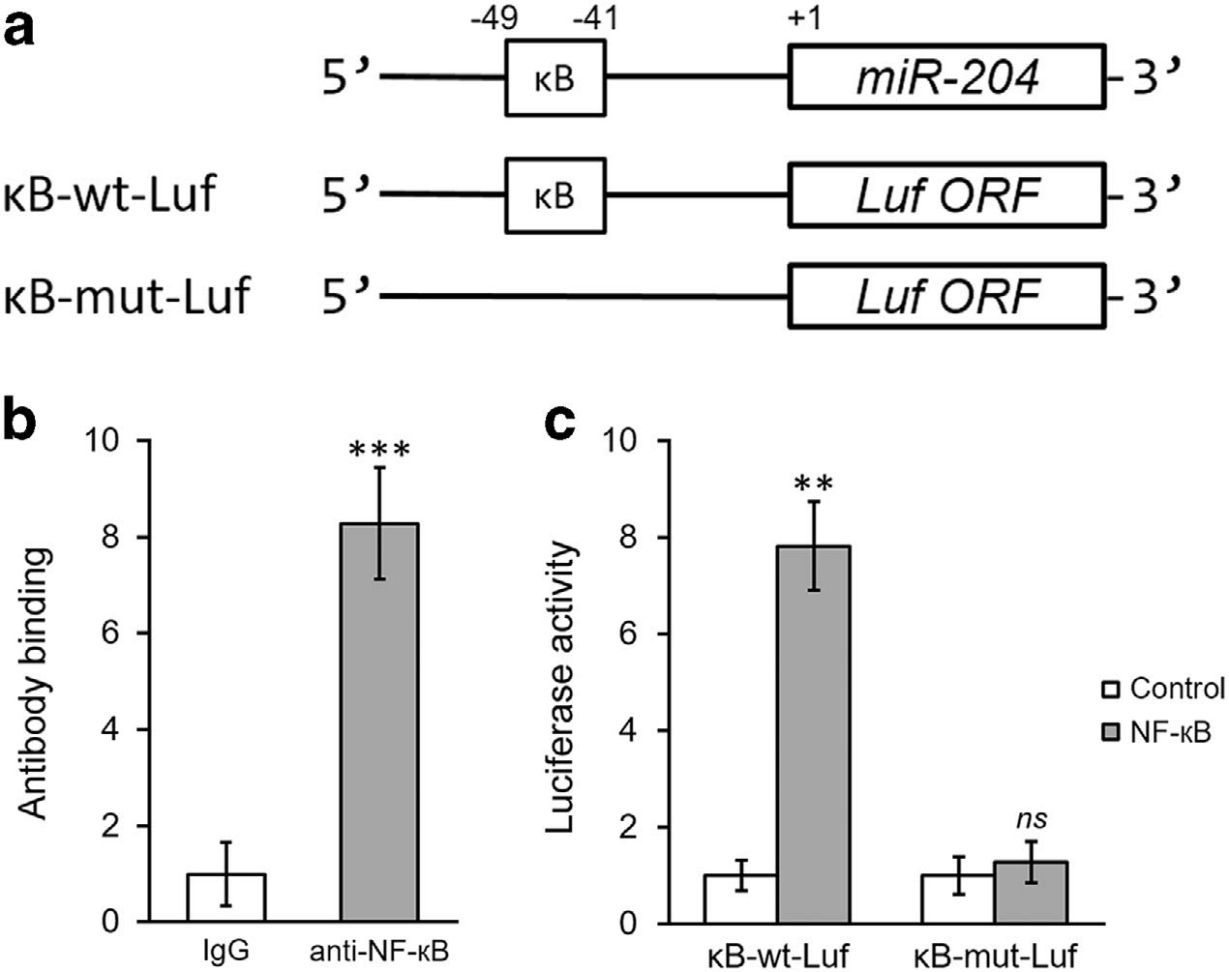
NF-κB directly binds to the promoter region of miR-204. (**a**) The putative NF-κB binding site in the promoter region of miR-204 (κB-wt-Luf) or a mutated version (κB-mut-Luf) was cloned into the upstream of a luciferase reporter gene (Luf ORF). (**b**) IgG control or specific antibodies against NF-κB were used in a Chromatin immunoprecipitation assay to analyze NF-κB binding to the promoter of miR-204 in hematopoetic stem cells (HSCs). (**c**) The luciferase activities of κB-wt-Luf and κB-mut-Luf constructs were measured in HSCs following control or NF-κB treatment. The values are shown as the mean±s.d. from three independent experiments. ***P<0.001 versus IgG. **P<0.01, NS, not significant, versus control.

As there is a putative NF-κB binding site (sequence: G/(T)GGRNNYYC/(T)C (N representing any base)) upstream (−41) of the *miR-204* promoter region (Figure 5a), we explored whether NF-κB could activate *miR-204* by directly binding to this region. Chromatin immunoprecipitation assays showed that NF-κB bound the *miR-204* promoter in HSCs, as shown by high enrichment of this region following p65 immunoprecipitation (IP) compared to precipitation with the control IgG (Figure 5b). Furthermore, two versions of the *miR-204* promoter region were cloned into luciferase reporter constructs, namely, κB-wt-Luf, containing the wild type sequence (Figure 5a), or κB-mut-Luf, containing a mutated NF-κB binding sequence (Figure 5a). As expected, NF-κB-stimulated luciferase activity in HSCs was only present in the κB-wt-Luf condition (Figure 5c). Taken together, our results suggested NF-кB-dependent transcriptional activation of *miR-204* may mediate TPO down-regulation, which can be reversed by IL-11 treatment in HSCs.

### IL-11 promotes HSC engraftment in an AA mouse model by inhibiting miR-204-5p

**Figure 6:**
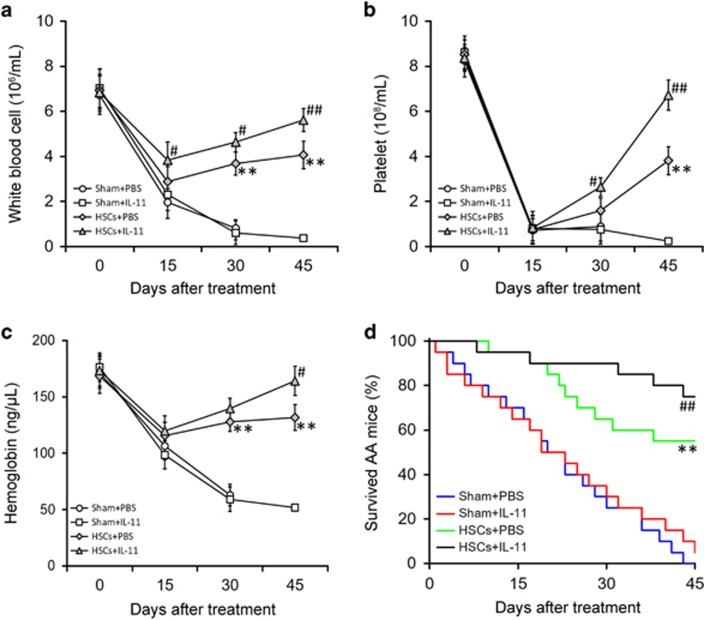
IL-11 treatment promotes treatment efficacy of hematopoetic stem cell (HSC) transplant in an AA mouse model. After establishing a mouse model of AA, HSC or sham transplants were performed (*n*=20), followed by PBS or IL-11 treatments. White blood cell count (**a**), platelet count (**b**), hemoglobin concentration (**c**) and survival rates (**d**) of each experimental group of mice were monitored for 45 days. The values are shown as the mean±s.d. **P<0.01 versus Sham+PBS and Sham+IL-11. ^##^*P*<0.01, ^#^*P*<0.05 versus Sham+PBS, Sham+IL-11 and HSCs+PBS.

To investigate the effects of IL-11 on AA *in vivo*, we administered IL-11 to mice in a benzene-induced model of AA.^31^ As shown in Figure 6, compared to sham controls (either with or without IL-11 treatment), mice with HSC transplantation gradually recovered their blood counts, including white blood cells (Figure 6a), platelet numbers (Figure 6b) and hemoglobin (Figure 6c). Notably, IL-11 administration significantly enhanced the efficiency of HSC engraftment (Figures 6a–c), eventually leading to an increased survival rate (Figure 6d).

**Figure 7:**
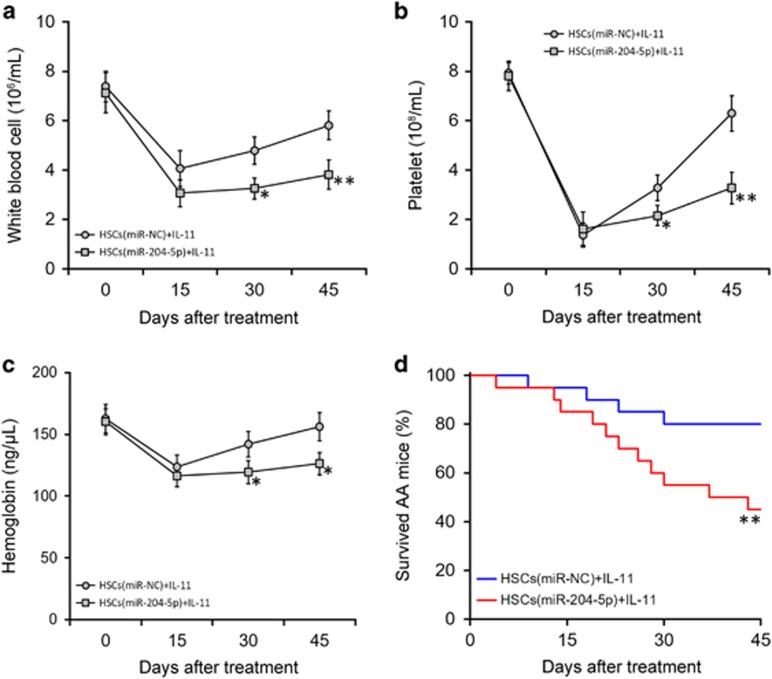
The promoting effect of IL-11 treatment on the efficacy of hematopoetic stem cell (HSC) transplant in an AA mouse model is mediated by the down-regulation of miR-204-5p. After establishing a mouse model of AA, HSCs stably expressing either miR-NC or miR-204-5p were transplanted into AA mice (*n*=20), followed by IL-11 treatment. The white blood cell count (**a**), platelet count (**b**), hemoglobin concentration (**c**) and survival rates (**d**) of each experimental group of mice were monitored for 45 days. The values are shown as the mean±s.d.. **P<0.01, *P<0.05, versus HSCs (miR-NC)+IL-11.

We finally examined whether the effects of IL-11 in treating AA mice were mediated through the suppression of miR-204-5p in HSCs. Control or miR-204-5p-overexpressing HSCs were transplanted to AA mice followed by IL-11 treatment. Blood counts results showed that miR-204-5p overexpression significantly impaired the role of IL-11 in HSC engraftment (Figures 7a–c). Accordingly, the survival rate of AA mice was significantly reduced following the transplantation of miR-204-5p-overexpressing HSCs compared with the transplantation of with control HSCs (Figure 7d). Thus, our data suggest that IL-11 treatment could promote BM transplantation in an AA mouse model via the repression of miR-204-5p. Of note, compared with control HSCs, the transplantation of TPO knockdown-HSCs showed significantly reduced efficacy in AA mice, and IL-11 treatment failed to improve this reduced efficacy (Supplementary Figure S1). These data clearly suggest that the promotion of HSC transplant efficacy by IL-11 was mediated entirely by TPO.

## Discussion

**Figure 8:**
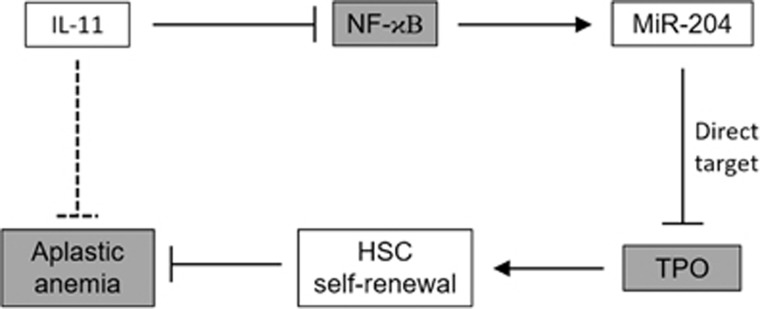
A working model illustrating the action of IL-11 in promoting the treatment efficacy of hematopoetic stem cell (HSC) transplant therapy against aplastic anemia. In HSCs, the anti-inflammatory cytokine IL-11 can inhibit NF-κB, which in turn represses miR-204 expression. miR-204-5p directly targets and inhibits expression of TPO, a positive regulator of HSC self-renewal. By repressing miR-204 levels in HSCs, IL-11 can enhance their self-renewal, thereby promoting their therapeutic efficacy against aplastic anemia following transplant.

We found that exposure to the anti-inflammatory cytokine IL-11 in mouse HSCs led directly to an inhibition of NF-κB signaling, which was critical for suppressing miR-204-5p expression. This effect was functionally essential for the induction of HSC proliferation by IL-11 given that miR-204-5p was found to target and repress the expression of TPO, a positive regulator of HSC self-renewal. Furthermore, the *in vivo* therapeutic efficacy of HSC transplantation in a mouse model of AA was enhanced by IL-11 treatment, an effect that was mediated by repressing miR-204-5p. This result indicates that a microRNA-dependent mechanism in HSCs underlies the therapeutic potential of IL-11 in AA (Figure 8).

miRNAs, as a group of gene expression regulators, are ∼22 nucleotide single-stranded non-coding RNAs that silence endogenous mRNA transcripts.^32^ HSC homeostasis is achieved through various intracellular pathways that include such small regulatory RNAs.^33, 34, 35, 36^ The genetic ablation of Dicer, the essential miRNA-processing enzyme, has revealed a cell-autonomous dependence of HSC populations on miRNAs.^34^ Being preferentially expressed in long-term HSCs, miR-125a can induce hematopoiesis expansion by increasing the numbers of HSC,^34^ while miR-125b is capable of maintaining lymphoid-balanced and lymphoid-biased HSC pools.^35^ Conversely, the forced expression of miR-126 in HSCs restrains cell-cycle progression, decreasing HSC numbers.^36^ As in many diseases, the roles of miRNAs in AA are considered potential targets for therapeutic interventions. The down-regulation of several miRNAs has been observed in the CD4^+^ and CD8^+^ T cells of patients with AA, potentially indicating a pathological role of T cell activation in this disease.^37^ More recently, both circulating plasma miRNAs^38^ and CD3^+^ T cell-associated miRNAs^39^ were evaluated in patients with acquired AA to identify potentially disease-specific miRNAs. Similar to our study, Zhao *et al.*^40^ suggested that miR-204 plays an essential role in the treatment of AA. Consistent with our current observations, these authors reported that decreasing miR-204 expression, which appeared to be responsible for the clinical efficacy of arsenic trioxide, might be a novel strategy in treating patients with AA.^40^ The investigations of this group have focused on bone marrow mesenchymal stem cells, which are important components of the bone marrow hematopoietic microenvironment. Taken together with our current HSCs-based study, these findings strongly demonstrate that miR-204 can salvage marrow failure, supporting the role of miR-204 in the therapeutic strategy of AA. On the other hand, the functional relevance of miR-204 in the pathogenesis of AA remains poorly understood. miR-204 was found associated with SOX2 and NANOG,^41^ two important stem cell transcription factors. In glioma cells and neural stem cells, miR-204 expression can inhibit stem cell-like phenotypes, such as self-renewal and invasion.^42^ However, the role of miR-204 in HSCs is unclear. Future studies should determine the extent of the roles of miR-204 in HSCs and evaluate whether the expression of this miRNA is altered in HSCs or other relevant cell populations during AA progression. It is possible that miR-204 expression is induced in response to unknown factors, contributing to the dysfunctions of HSCs in AA by suppressing the self-renewal, proliferation, differentiation and migration of hematopoietic progenitors.

Mechanistically, our data support a model in which NF-κB-dependent miR-204-5p expression can be repressed by IL-11. NF-κB signaling, which has been shown to induce the expression of various miRNAs,^43^ is a dominant driver of the Th1-dependent immune response. As such, it is assumed that NF-κB activation plays an indispensable role in the development of bone marrow failure in AA.^44^ The finding of NF-κB-dependent TPO repression through miR-204-5p provides a novel insight into the known negative effects of pro-inflammatory cytokines in HSCs. TPO signaling primarily mediates megakaryocyte growth and development, and recent studies have also identified its functions in maintaining the quiescence and self-renewal of HSCs.^45^ It has been reported that TPO can promote HSC quiescence during adult hematopoiesis, and decreased TPO signaling is associated with marrow failure or thrombocytopenia.^45^ Our results suggest a beneficial role of TPO induction in promoting HSC engraftment in a mouse AA model. Further work is required to determine whether TPO up-regulation is the sole reason responsible for the effects of IL-11 in HSCs and how TPO induction may constitute a useful treatment for AA.

Finally, our data are consistent with the model in which anti-inflammatory cytokines, such as IL-11, are capable of reversing marrow dysfunction in diseases such as AA. Recombinant IL-11 has been used to diminish activated Th1 cells,^19^ and its thrombopoietic activity was reported in patients with bone marrow failure caused by myelodysplastic syndromes, graft failure, chemotherapy and AA.^20, 21^ The effects we observed in the present study seem to be mediated by an autonomous signaling pathway in HSCs. Via a known inhibitory effect on NF-κB signaling, IL-11 was shown to support the self-renewal of HSCs isolated from mouse bone marrow. Although we specifically investigated the direct contribution of IL-11 signaling to HSC proliferation *in vitro*, the *in vivo* results might be complicated by other environmental factors. As benzene toxicity appears to primarily affect hematopoietic cell survival, the finding that IL-11 promotes HSC engraftment in benzene-induced bone marrow failure was consistent with the *in vitro* effects of IL-11 on HSC proliferation. However, benzene hematotoxicity also affects bone marrow stromal cells, reducing their ability to support hematopoietic cell growth and survival via cytokines. Thus, the rescued hematopoiesis in the IL-11-treated AA mouse model could also be explained by its ameliorating effects on the immune response. Thus, it is of importance to dissect these effects in further studies, for example, by addressing the impacts of IL-11 on HSC niche compartments.

In conclusion, IL-11 stimulates HSC proliferation and increases TPO expression *in vitro*, which may explain the stimulating role of IL-11 treatment on HSC transplantation in a mouse model of aplastic anemia. This IL-11 function relies on a suppression of NF-κB-dependent miR-204-5p expression in HSCs, which in turn negatively regulates TPO levels. The data collectively support that IL-11-induced miR-204-5p reduction may be a therapeutic strategy in patients with AA who require HSC transplantation.
