# Supplementary material for: IL-11 promotes the treatment efficacy of hematopoietic stem cell transplant therapy in aplastic anemia model mice through a NF-κB/microRNA-204/thrombopoietin regulatory axis
Source: Exp Mol Med. 2017 Dec 8;49(12):e410–. doi: 10.1038/emm.2017.217 (PMC5750475; doi:10.1038/emm.2017.217)
Supplement: Supplementary Figure S1 [file emm2017217x1.docx]

**Supplementary Materials**

**
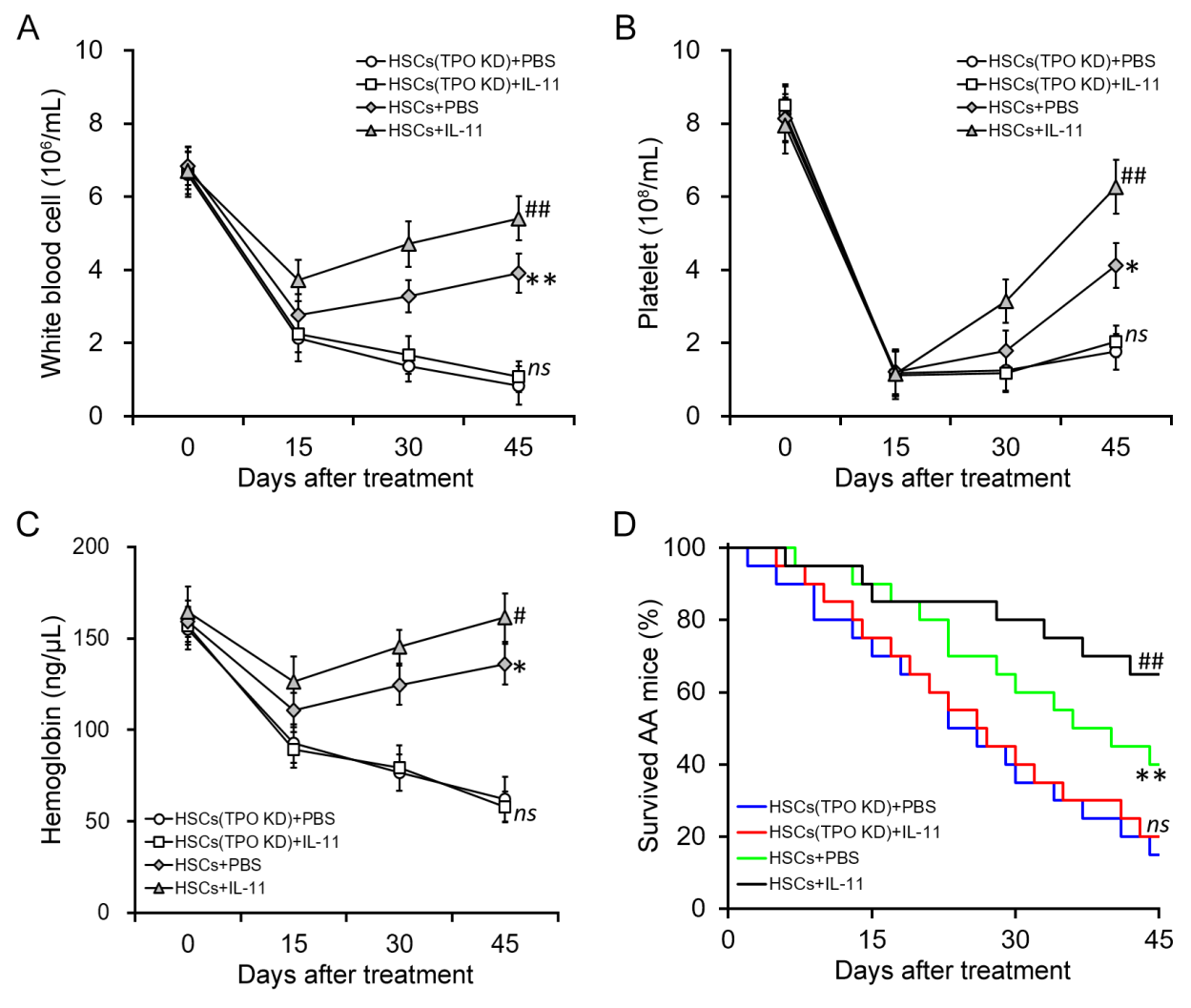
**

**Supplementary Figure S1. Promotion of HSC transplant efficacy by IL-11 is mediated entirely by TPO.**

After establishing a mouse model of AA, transplant using either HSC or HSC with TPO knockdown (KD) were performed in the AA mice (n=20), followed by PBS or IL-11 treatments, respectively as indicated. White blood cell count (A), platelet count (B), hemoglobin concentration (C) and survival rates (D) of each experimental group of mice were monitored for 45 days. Values were shown as mean ± SD. ** P < 0.01, * P < 0.05 versus HSCs(TPO KD)+PBS and HSCs(TPO KD)+IL-11. ## P < 0.01, # P < 0.05 versus HSCs(TPO KD)+PBS, HSCs(TPO KD)+IL-11 and HSCs+PBS. *ns* not significant, versus HSCs(TPO KD)+PBS.
